# Phenotype-driven chemical screening in zebrafish for compounds that inhibit collective cell migration identifies multiple pathways potentially involved in metastatic invasion

**DOI:** 10.1242/dmm.018689

**Published:** 2015-06-01

**Authors:** Viviana E. Gallardo, Gaurav K. Varshney, Minnkyong Lee, Sujata Bupp, Lisha Xu, Paul Shinn, Nigel P. Crawford, James Inglese, Shawn M. Burgess

**Affiliations:** ^1^ Developmental Genomics Section, Genome Technology Branch National Human Genome Research Institute, National Institutes of Health, Bethesda, MD 20892, USA; ^2^ Genetics and Molecular Biology Branch, National Human Genome Research Institute, National Institutes of Health, Bethesda, MD 20892, USA; ^3^ Department of Pre-Clinical Innovation, National Center for Advancing Translational Sciences, National Institutes of Health, Rockville, MD 20850, USA

**Keywords:** Drug screening, Metastasis, Orthotopic implantation, Src, Tks5, Zebrafish

## Abstract

In the last decade, high-throughput chemical screening has become the dominant approach for discovering novel compounds with therapeutic properties. Automated screening using *in vitro* or cultured cell assays have yielded thousands of candidate drugs for a variety of biological targets, but these approaches have not resulted in an increase in drug discovery despite major increases in expenditures. In contrast, phenotype-driven screens have shown a much stronger success rate, which is why we developed an *in vivo* assay using transgenic zebrafish with a GFP-marked migrating posterior lateral line primordium (PLLp) to identify compounds that influence collective cell migration. We then conducted a high-throughput screen using a compound library of 2160 annotated bioactive synthetic compounds and 800 natural products to identify molecules that block normal PLLp migration. We identified 165 compounds that interfere with primordium migration without overt toxicity *in vivo*. Selected compounds were confirmed in their migration-blocking activity by using additional assays for cell migration. We then proved the screen to be successful in identifying anti-metastatic compounds active *in vivo* by performing orthotopic tumor implantation assays in mice. We demonstrated that the Src inhibitor SU6656, identified in our screen, can be used to suppress the metastatic capacity of a highly aggressive mammary tumor cell line. Finally, we used CRISPR/*Cas9*-targeted mutagenesis in zebrafish to genetically validate predicted targets of compounds. This approach demonstrates that the migrating PLLp in zebrafish can be used for large-scale, high-throughput screening for compounds that inhibit collective cell migration and, potentially, anti-metastatic compounds.

## Abstract (precis)

**Summary:** We have developed a phenotype-driven screen for identifying new inhibitors of collective cell migration and demonstrated the screen can successfully identify compounds active *in vivo* and potentially new pathways for targeting cancer metastasis.

## INTRODUCTION

Cancer primarily kills by metastasis, with at least 90% of cancer deaths being caused not by the primary tumor but by cancer invasion in remote tissues. Yet, metastasis remains the most poorly understood component of cancer pathogenesis because this process is inherently difficult to model and study *in vitro*. The transition of a cancer from a ‘localized’ tumor to a metastatic form requires the acquisition of several cellular transformations, including migratory propensity and invasiveness (Chaffer and Weinberg, 2011; Valastyan and Weinberg, 2011), and these transformations are strongly influenced by the surrounding normal, heterogeneous tissues.

High-throughput screens using *in vitro* or cell-based models that typically target specific candidate genetic pathways have been developed to identify drugs that can inhibit collective cell migration in cancer metastasis (Chua et al., 2012; Quintavalle et al., 2011). These studies generated many ‘hits’; however, recent analysis has demonstrated that target-based screening has a very poor success rate when it comes to identifying potential therapeutic drugs (Swinney and Anthony, 2011). In contrast, phenotype-driven screening has a much higher rate of success (Swinney and Anthony, 2011); therefore, the closer one can model the ‘natural’ environment of cell migration *in vivo*, the more likely it is one will discover novel compounds with potential therapeutic value.

Tissue opacity in most animal model systems makes real-time studies of collective cell migration in morphogenesis and cancer difficult. However, using transgenics and time-lapse imaging, the migration of the zebrafish posterior lateral line primordium (PLLp) has recently emerged as a powerful model to investigate the molecular mechanisms and regulation of collective cell migration (Aman and Piotrowski, 2010; Friedl and Gilmour, 2009). The PLLp is a group of migrating cells that moves dorsally along the body of the fish, depositing clusters of cells along the way that become the lateral line neuromasts (Aman and Piotrowski, 2011; Ghysen and Dambly-Chaudiere, 2007). Parallels can been drawn between the collective migration of the PLLp cells and the behavior of invasive cancer cells. In both events, cells delaminate, acquire migratory behavior, and progress through extracellular matrix to reach a distant target.

Our previous studies showed that a large number of genes expressed specifically in the migrating primordium have common roles in collective cell migration and cancer progression (Gallardo et al., 2010). In addition, others have shown that the main signaling pathways responsible for the regulation of cell mobilization are active during both development and tumor metastasis (Yang and Weinberg, 2008). Therefore, it is possible that compounds that inhibit the natural progression of the posterior lateral line (PLL) also have potent anti-metastatic activity.

Given the cellular and molecular parallels between lateral line development and metastasis, we developed a whole-organism-based chemical screening strategy combined with automated fluorescence microscopy to identify small-molecule modulators of zebrafish lateral line migration. We took advantage of the transgenic reporter line *cldnb:EGFP* (Haas and Gilmour, 2006), which expresses GFP in all of the cells of the PLL and, using this reporter line, screened a collection of drugs and other bioactive compounds (Sigma LOPAC 1280), a collection of 800 natural products (NatProd Collection), and the GSK Published Kinase Inhibitor Set (PKIS) to identify compounds that inhibited collective cell migration. We identified 165 compounds that interfered with primordium migration without overt toxicity *in vivo*. We identified several kinase inhibitors, flavonoid-derivatives and antioxidants that all disrupted primordium migration. We also confirmed, in a mouse tumor model, that the Src inhibitor SU6656 also specifically decreases tumor metastasis. Finally, we showed that target pathways can be quickly genetically validated in zebrafish by using CRISPR/*Cas9* targeting. Taken together, our approach suggests that the migrating PLLp in zebrafish can be used for large-scale, high-throughput screening for inhibitors of collective cell migration.

**TRANSLATIONAL IMPACT**

**Clinical issue**

Cancer is a leading cause of death worldwide. As high as 90% of cancer deaths are a result of metastasis, yet this remains the most poorly understood component of cancer pathogenesis. The current preclinical pipeline for target-driven drug discovery involves multiple rounds of *in vitro* biochemical and cell-based assays followed by *in vivo* studies in animal models, and finally trials in humans. This process typically takes 12-15 years before drugs reach the market and is expensive, limiting the number of compounds that can effectively be translated into therapeutic use. Over the past decade, the focus of drug screens has been on high-throughput screens using *in vitro* assays or cell-based models that target specific candidate pathways, with the aim of inhibiting cancer metastasis. These studies have generated thousands of candidate drugs for a variety of biological targets; however, these approaches have had very poor success rates when it came to therapeutic drugs because they generally lacked relevant whole-organism physiology. Most of the positive *in vitro* results were not replicated when tested *in vivo*. Thus, an *in vivo* phenotype-driven screen in a whole-animal model should provide better targets for therapeutic intervention with a much stronger success rate, shortening years of research and increasing cost-effectiveness.

**Results**

In this study, the authors developed a robust *in vivo* assay using transgenic zebrafish to mark the migrating posterior lateral line primordium as readout for inhibition of collective cell migration. Via a high-throughput screening protocol, the authors identified a number of compounds, which included novel flavonoid-derivative molecules and a cluster of structurally related kinase inhibitors that interfered with primordium migration without overt toxicity *in vivo*. The goal of identifying cell-migration inhibitors is to find compounds that have anti-metastatic activity. Demonstrating the utility of this approach, the authors confirmed, by performing orthotopic tumor implantation assays in mice, that inhibition of the Src pathway decreases tumor metastasis *in vivo*. Finally, the authors used CRISPR/*Cas9* targeted mutagenesis in zebrafish to validate targets of essential genes involved in cell migration, showing that zebrafish can be used to rapidly confirm the molecular targets of inhibitory compounds.

**Implications and future directions**

This study highlights the utility of the zebrafish migrating primordium as an *in vivo* large-scale, high-throughput screening system for cell-migration inhibitors. This study also demonstrates that this screen can be used to successfully identify both compounds and new pathways for targeting cancer metastasis. In addition, this approach represents a starting point for future in-depth studies to develop new therapeutic strategies for cancer.

## RESULTS

### Screening for cell-migration inhibitors

We developed a whole-organism-based chemical screening strategy to rapidly identify novel small-molecule modulators of cell migration during zebrafish PLL formation. We used *cldnb:EGFP* embryos to screen the LOPAC 1280 library, the PKIS and the NatProd collection for compounds that alter the migration of the lateral line.

**Fig. 1.**
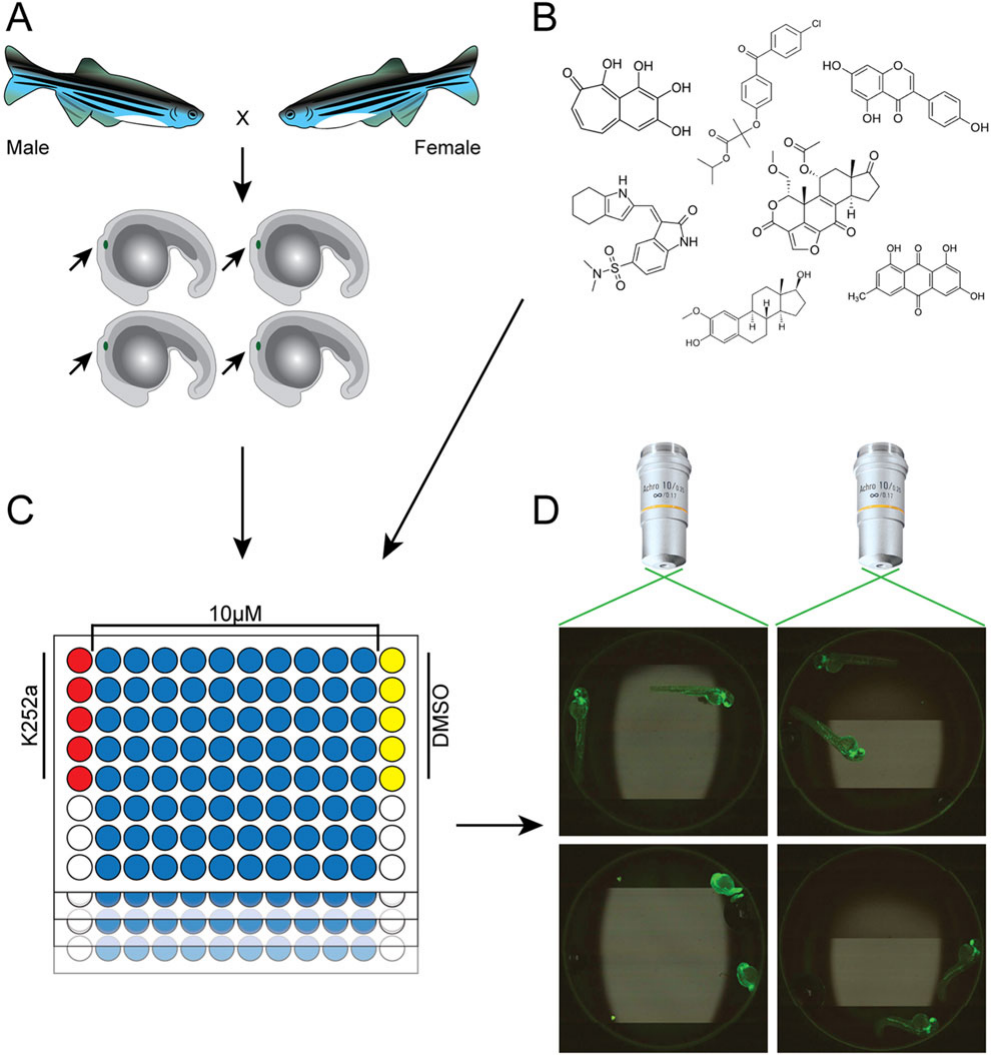
**Overview of the *in-vivo* drug screening strategy in zebrafish.** (A) The LOPAC1280, NatProd and PKIS libraries were screened for cell-migration inhibitors using 20 hpf *cldnb:EGFP* embryos that were GFP-positive for the migrating PLLp (marked with small arrow). (B) Compounds were transferred by ultrasonic dispenser from 384-well plates to 96-well plates so that, when 200 µl of embryo medium was added, the compounds were at a final concentration of 10 μM (NCATS, NIH). (C) Each plate contained five negative (1% DMSO) and five positive (0.01-1 μM K252a) control wells. Two embryos were placed manually into each well of the 96-well plates and PLL development was followed through 48 hpf. (D) Automated image capture of zebrafish embryos was performed using an iCys^®^ Research Imaging Cytometer and images were scored for completion of PLLp migration.

At 20 h post-fertilization (hpf) (which coincides with the onset of the primordium migration), *cldnb:EGFP* embryos were manually arrayed into 96-well dishes (two embryos per well) using a 200-µl wide-bore pipette tip and treated with test compounds at a final concentration of 10 μM. All plates contained five negative control wells (1% DMSO) and five positive control wells (K252a, the broad activity kinase inhibitor previously determined to arrest PLLp migration) (Fig. 1), and migration for each compound was scored in comparison to the control wells.

Embryos were monitored over a period of 2 days for correct lateral line migration. A fluorescence image of each well was captured by using an automated imaging system at 16 and 28 h post-incubation with the test compounds (Fig. 1D). In addition to migration disruption, drug-induced lethality and other visible phenotypes were scored. We identified all PLLp cells that had not migrated to the tail of the fish by 48 hpf. Compounds that scored positive for inhibiting migration were retested and all compounds that scored positively twice were considered as potentially anti-metastatic compounds.

The majority (1543 compounds, 74.18%) of the 2080 compounds tested from the LOPAC and NatProd libraries did not cause an observed phenotype in 2-dpf *cldnb:EGFP* embryos. Of the remaining compounds, 21% were toxic at 10 µM. We retested the toxic compounds at final concentrations of 1 and 5 μM. On the other hand, more than 90% of compounds initially tested with the PKIS were toxic at 10 µM. Consequently, we screened this library at 0.5, 1 and 5 µM.

**Fig. 2.**
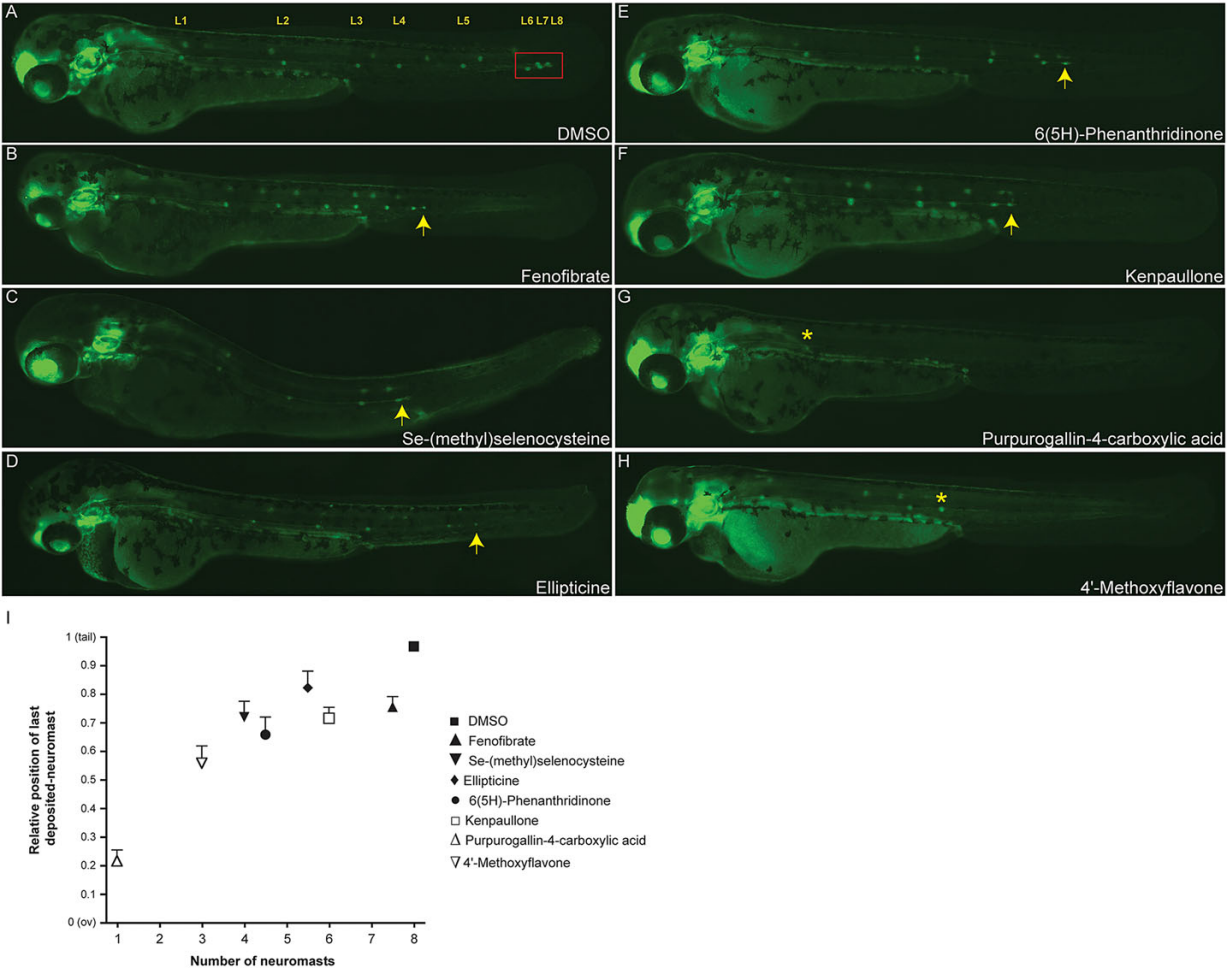
**Examples of PLL migration phenotypes observed in the screen.** Phenotypes of 48 hpf zebrafish embryos treated with small molecules that had been identified in the screen. (A) DMSO control. The red box indicates the successful migration of the terminal neuromasts to the end of the tail. *Cldnb:EGFP* embryos treated with 10 μM of Se-(methyl)selenocysteine (C), Ellipticine (D) or 6(5H)-Phenanthridinone (E) show a delay in primordium migration and, therefore, have disrupted lateral line formation compared with the DMSO control (A,I), whereas treatment with Fenofibrate (B) or Kenpaullone (F) causes an increase in the neuromast deposition rate, resulting in a phenotype with premature deposition of terminal neuromasts. Treatments with the flavonoid-derivative molecules Purpurogallin-4-carboxylic acid (G) and 4′-Methoxyflavone (H), almost completely abolished the posterior lateral line formation, generating PLL phenotypes of 1 (G) and 3 (H) deposited neuromasts, respectively. Arrows indicate the position of the PLLp relative to the fully successful migration marked by the red box in A; asterisks indicate the position of the last deposited neuromast in G and H. (I) Quantification of the average position of the final deposited neuromast in drug-treated embryos relative to the distance from the otic vesicle (ov) to the tip of the tail. Experiments were carried out using 15 larvae per condition. Error bars indicate +s.d. Compounds showed a variety of effects, from inhibiting both neuromast numbers and migratory behavior (e.g. Purpurogallin-4-carboxylic acid) to only slowing migration (Fenofibrate).

Altogether we identified 165 compounds that interfered with primordium migration without causing overt toxicity (gross developmental defects) in the larvae. The screen resulted in a compound list of activity annotations related mainly to phosphorylation, neurotransmission and cell signaling. Overall, 5.57% of compounds screened disrupted collective migration of the primordium and, therefore, the development of the PLL. Fig. 2 shows representative PLL phenotypes observed in the primary screen. Exposure to a variety of inhibitor classes resulted in disruption of migration. For example, the PPAR-alpha receptor agonist Fenofibrate (Fig. 2B) and the cyclin-dependent kinase (CDK) inhibitor Kenpaullone (Fig. 2F) induce a phenotype with the premature deposition of a terminal neuromasts, whereas treatments with the cell-cycle inhibitor methylselenocysteine (Fig. 2C), the DNA-topoisomerase-II inhibitor Ellipticine (Fig. 2D) and the PARP inhibitor 6(5H)-Phenanthridinone (Fig. 2E), all disrupted lateral line migration compared with the DMSO controls (Fig. 2A). Treatment with the antioxidant Purpurogallin-4-carboxylic acid (Fig. 2G) or the flavonoid-derivative, 4′-Methoxyflavone (Fig. 2H) completely abolished the PLL. Quantitative analysis of the last deposited neuromast at 48 hpf is shown in Fig. 2I. Average position of deposited neuromast was estimated relative to the distance from the otic vesicle (ov) to the tip of the tail as performed in Matsuda et al. (2013).

Our screening identified a wide variety of pathways whose inhibition had potentially anti-metastatic effects (supplementary material Table S1). Additionally, the following six broad categories were targeted by two or more compounds: cell signaling, gene regulation, ion channels, lipid metabolism, neurotransmission and natural products of undefined activity. There were compounds with interesting phenotypes not specifically related to migration; for example, the two EGFR inhibitors Tyrphostin AG1478 and GW2974 generated supernumerary neuromast numbers unrelated to a migration phenotype (supplementary material Fig. S1), supporting the idea that ErbB signaling is required in glial cells to repress the precocious neuromast formation from cells that lay between neuromasts (Grant et al., 2005).

### Small-molecule compounds affect collective migration of the PLLp

To confirm the result of the primary screen we tested a group of 18 compounds, selecting the kinase-related molecules and the most active natural products. We grouped these compounds by the genes that they were designed to target (supplementary material Table S2). This kinase-compound list contained three Src-family kinase inhibitors: Emodin, SU6656 and 7-cyclopentyl-5-(4-phenoxy)phenyl-7H-pyrrolo[2,3-d]pyrimidin-4-ylamine (RBI); two PDGFR inhibitors, Tyrphostin A9 and SU 4312; and the CDK inhibitors Kenpaullone and Indirubin-3′-oxime, among others.

Five highly active compounds in the natural products collection were identified in the screen. The list contained three flavonoid-derivatives – genistein, 7,3′-dimethoxyflavone (previously identified as an anti-invasive compound by Parmar et al., 1994) and 4′-Methoxyflavone; the antioxidant Purpurogallin-4-carboxylic acid and the aldose-reductase inhibitor 4-Naphthalimidobutyric acid – all of which strongly inhibited the migration of the PLL.

### Identified compounds inhibit cell migration in other contexts

**Fig. 3.**
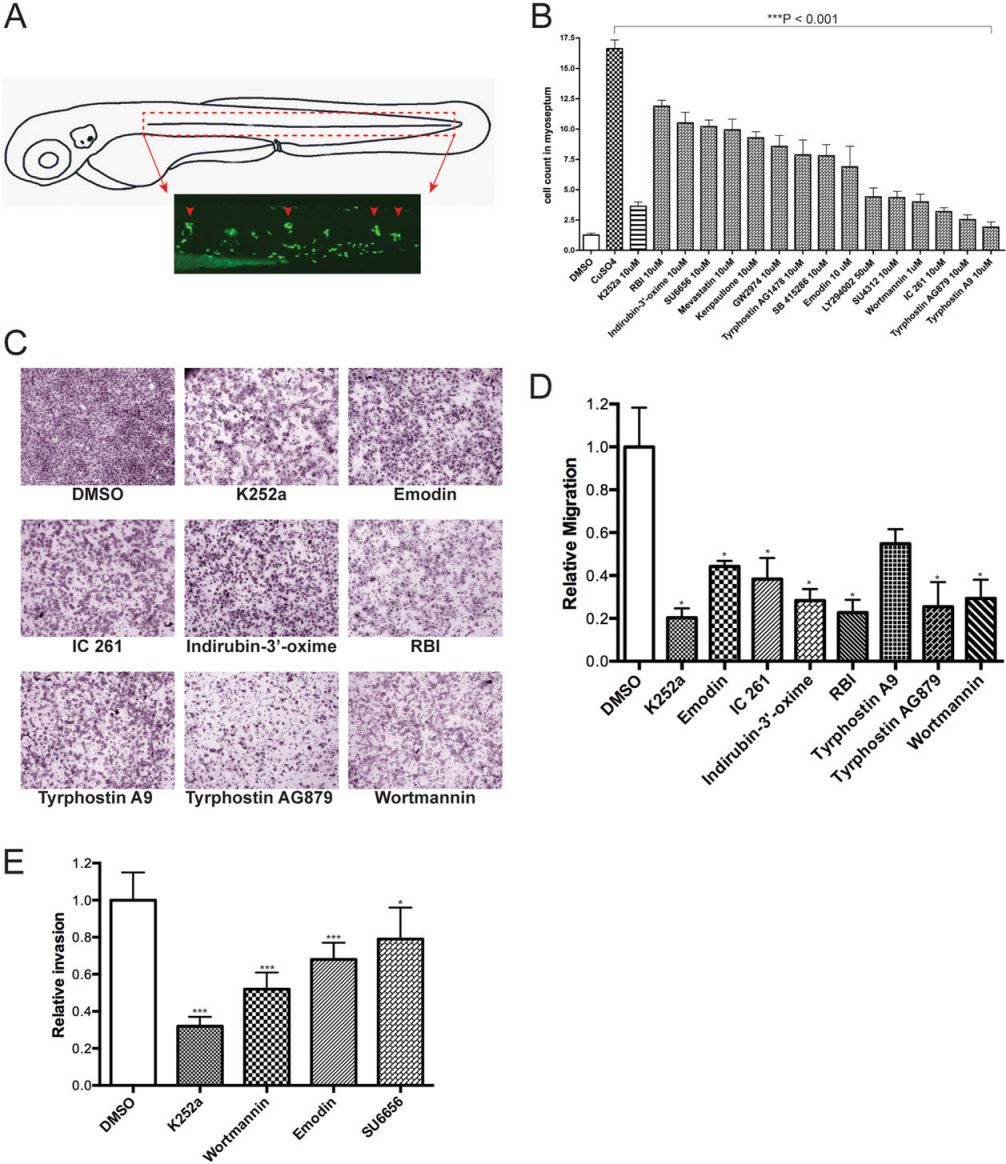
**Confirmation of active compounds.** (A) Schematic view of a 3 day post-fertilization (dpf) larva. The boxed area corresponds to the horizontal myoseptum (dotted red lines) and shows the area where leukocytes were counted in all quantification experiments. (B) Quantification of infiltrating leukocytes in the lateral line after treatments with diverse inhibitors. The assays were carried out by manually counting leukocytes recruited to the lateral line neuromasts after Cu^2+^ treatment (10 μM) as described in Results. In this experiment, drugs were added 30 min prior to Cu^2+^ treatment. The graph shows average leukocyte numbers in the lateral line in negative controls (DMSO), in Cu^2+^ (as CuSO_4_) and in Cu^2+^-plus drug-treated fish. Leukocyte quantification shows significant reduction (***P<0.001) in neutrophil recruitment to damaged PLL neuromasts in larvae exposed to the compounds tested compared with Cu^2+^-exposed larvae, suggesting an inhibition of the neutrophil migration. Experiments were carried out with 15 larvae per condition. (C) Effects of kinase inhibitor compounds from LOPAC1280 on *in vitro* melanoma cell migration/invasion. 501-mel human melanoma cells were treated with DMSO (negative control) or the c-Met inhibitor K252a (positive control), and with the drugs indicated for 16 h and then assayed for motility as described in Materials and Methods. Representative data from independent experiments are shown. Only compounds that had a negative effect on migration are shown. (D) Quantification of 501-mel cell migration assays. The migratory cells were counted and the results expressed as the mean number of migratory cells from two independent experiments. Measurements were calculated relative to control (DMSO). (E) Comparative effects of tested drugs on the cell invasion capacity of melanoma cells. 501-mel cells were treated with DMSO, K252a, Wortmannin, Emodin and SU6656 and then plated on Matrigel inserts in presence of HGF at 50 ng/ml for 24 h. The invasive cells were counted and the results expressed as the mean number of migratory cells in five random microscopic fields. Measurements were calculated relative to control (DMSO). Data collected were from two independent experiments. ***P<0.001; *0.01<*P*<0.05.

To provide independent verification that we are identifying compounds that inhibit cell migration, we performed an *in vivo* neutrophil migration assay in zebrafish, in which specific non-invasive damage to the lateral line neuromasts by using Cu^2+^ (in the form of CuSO_4_) induces an acute inflammatory response (Fig. 3A) (d'Alencon et al., 2010). Compounds that inhibit cell migration would prevent the invasion of neutrophils into the damaged lateral line neuromasts. We used the *Tg(mpx:GFP)* transgenic fish, which allowed us to track tissue damage by visual observation of GFP expressed in the neutrophils. We exposed 72 hpf *Tg(mpx:GFP)* larvae to the 15 most effective (based on arrest strength and least toxicity) inhibitors of the LOPAC1280 library (supplementary material Fig. S2) and four compounds of the NatProd library (supplementary material Table S3) for 30 min to allow the penetration of the drugs into larval tissues, followed by the addition of Cu^2+^ for 40 min. As expected, the DMSO controls showed the normal distribution of neutrophils primarily localized in the ventral trunk and tail. However, in Cu^2+^-treated larvae, neutrophils clustered around the neuromasts as part of the inflammatory response. In drug-treated larvae, all of the selected compounds tested, including the positive controls of K252a, exhibited statistically significant inhibition – on the basis of neutrophil cell counts – within a defined region surrounding the neuromast (supplementary material Fig. S2). In contrast, larvae pre-treated with hydrocortisone (a compound that scored negative in the PLLp assay) did not exhibit significant inhibition in chemically induced inflammation (ChIn) assays (data not shown), as previously described in d'Alencon et al., 2010. Quantitative analysis for a subset of tested drugs is shown in Fig. 3B.

### Comparison of compounds identified by *in vivo* screening with traditional *in vitro* cell-based metastasis models

To further explore the generality of our results, we tested the effect of the selected compounds in *in vitro* cell-migration or cell-invasion assays by using melanoma-derived cell lines. We tested human 501-mel melanoma cells with kinase inhibitors from the LOPAC1280 library for further evaluation. Eleven of the compounds caused significant inhibition of cell migration. The remaining compounds did not show significant inhibition compared to the DMSO control. Assays were repeated three times and representative pictures are shown in Fig. 3C. Quantification of the cell migration assay is shown in Fig. 3D. For invasion assays, we used hepatocyte growth factor (HGF) to induce migration, as it has been shown to stimulate the invasive potential of melanoma cells (McGill et al., 2006). For Matrigel invasion assays, we observed that treatment of 501-mel cells with the tested drugs resulted in inhibition of invasion of these cells induced by HGF. Quantification of four compounds, K252a (a broad kinase inhibitor), Wartmannin (PI3K), Emodin (Src) and SU6566 (Src), is shown in Fig. 3E.

Using Trypan Blue, we verified that treatment of 501-mel cells had no significant effect on cell viability for any of the tested drugs (data not shown). Together, these results confirmed many of the candidate drugs from our screen work when exposed to traditional cell-based cancer invasion models. More importantly, we were able to identify many compounds that worked *in vivo*, but would not have been identified in a cell-based screen (supplementary material Table S3).

### Using CRISPR/*Cas9* in zebrafish embryos to rapidly validate inhibitor targets

**Fig. 4.**
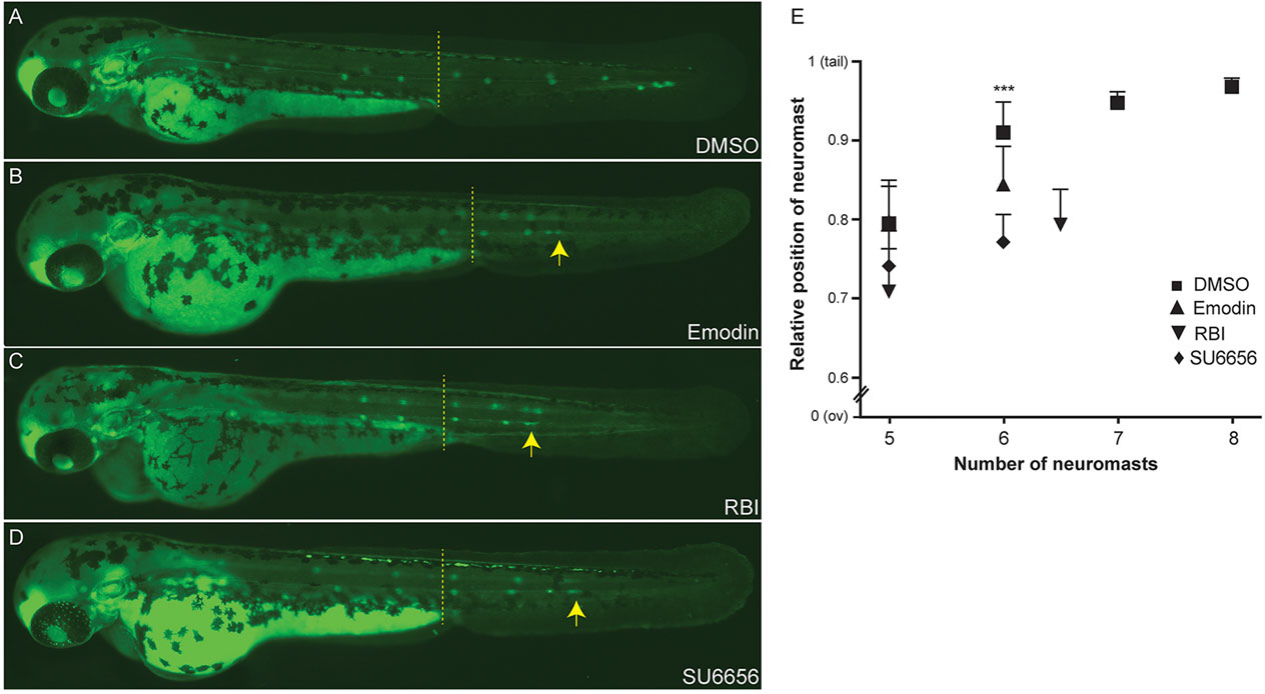
**Blocking the Src signaling pathway inhibits collective cell migration *in vivo*.** *Cldnb:EGFP* embryos were treated at 36 hpf (primordium has migrated beyond the yolk extension, dashed line) with the c-Src inhibitors Emodin (B), RBI (C) or SU6656 (D). Treated embryos show a disruption of the primordium migratory ability compared with DMSO control (A), resulting in a phenotype with a premature deposition of a terminal neuromasts (L6) in all conditions (B-D). (E) Quantification of average position of the last five deposited neuromasts in drug treated-embryos relative to the distance from the ov to the tip of the tail. Arrows indicate the position of the PLLp. Experiments were carried out with ten larvae per condition. Error bars indicate +s.d.; ***P<0.001.

**Fig. 5.**
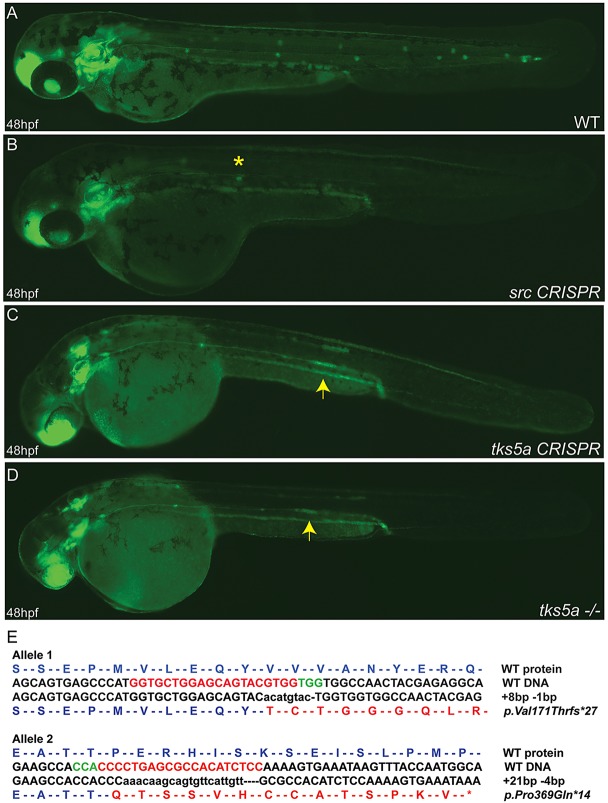
**Disruption of *src* and *tks5a* genes by CRISPR/*Cas9* affects the PLLp migration.** Two sgRNAs targeting two different exons (25 ng/µl) within each gene were co-injected with Cas9 mRNA (300 ng/µl) into *cldnb:EGFP* embryos and the embryos raised. Injected fish were inbred and at 48 hpf WT embryos show the normal pattern of the migrating primordium and neuromasts deposition (A), whereas *src* CRISPR (B), *tks5a* CRISPR (C) and *tks5*a^−/−^ (D) embryos show a disruption of the primordium migratory ability and PLL morphogenesis. Arrows indicate the position of the primordium in *tks5a^−/−^* embryos; asterisk indicates the position of the last deposited neuromast. (E) Sequence confirmation of *tks5a^−/−^*alleles. Allele 1 has an 8-bp insertion and a 1-bp deletion, which changed amino acid valine at position 171 to threonine and truncates the protein after 27 amino acids. Allele 2 has a 21-bp insertion and a 4-bp deletion, which changed amino acid proline at position 369 to glutamine and truncated the protein after 14 amino acids.

The Src-Tks5 pathway has been demonstrated to be involved in the regulation of the migration of neural crest cells in zebrafish (Murphy et al., 2011), in macrophage invasive behavior (Burger et al., 2011) and in cancer cell invasion (Blouw et al., 2008; Courtneidge, 2012). Not surprisingly, we identified in our screening three Src inhibitors (supplementary material Table S1) that interfere with PLLp migration. When tested compounds were added either at 20 hpf (when primordium migration begins; data not shown) or 36 hpf (when primordium migrated beyond the yolk extension and 2-3 neuromasts have already been deposited), we observed in all cases a disruption in the migration of the primordium compared to the DMSO control (Fig. 4). As a result, Emodin-, RBI- and SU6656-treated embryos lacked the most caudal PLL neuromasts. Typically, embryos had only the first five or six neuromasts, with closer deposition of the neuromasts (Fig. 4E). Although the Src pathway has been widely implicated in diverse cellular processes, Src activity has neither been shown to be involved in PLLp migration during development nor have Src inhibitors been shown to disrupt this process. Since the three Src inhibitors had been independently identified in the screen, evidence is strong that Src is the true target; however, one major consideration when screening inhibitors is the significant issue of ‘off-target’ effects for many of the compounds. Morpholinos have a known artifact that impacts lateral line migration, precluding their use, but CRISPR/*Cas9* has been shown to be a very effective technique for targeting genes in zebrafish (Hwang et al., 2013; Jao et al., 2013). To confirm that it was truly inhibition of the Src pathway that impacted lateral line migration, we used CRISPR/*Cas9* to target *src* or a downstream substrate of *src*, *tks5a*. When single-guide RNAs (sgRNAs) targeting *src* and *tks5a* (but not unrelated control sgRNAs) were injected into the *cldnb:EGFP* transgenic line, lateral line migration was blocked in a manner similar to that of Src inhibitors or morpholinos targeting *tks5a* (data not shown) (Murphy et al., 2011). At 48 hpf, embryos injected with CRISPR sgRNAs targeting *src* showed a gross disruption of primordium migration (Fig. 5B). Src-mutant embryos showed strong migratory inhibition and deposition of very few – typically one or two – neuromasts. We observed a similar but less-severe phenotype in *tks5a*-CRISPR-injected embryos (F_0_) and *tks5a^−/−^* F_1_ homozygous mutants (Fig. 5C,D). At 48 hpf, *tks5a^−/−^* embryos showed delayed migration of the primordium and PLL morphogenesis. Other defects were also visible in mutants, such as: smaller heads with small eyes, edema and overt delay in appearance of pigment cells in the tail (Murphy et al., 2011). Compound heterozygous *tks5a* mutants contained small indels in exon 8 and exon 12. Exon 8 carried an 8-bp insertion and 1-bp deletion, whereas exon 12 carried a 21-bp insertion and 4-bp deletion (Fig. 5E). The more-drastic phenotype observed in *src*-CRISPR-injected embryos compared with *tks5a^−/−^* mutants could be because Src is located upstream of Tks5, regulating other biological pathways beyond those directly utilizing the *tks5a* gene. This demonstrates how quickly candidate targets can be confirmed (or identified in the case of multiple known targets of an inhibitor) in zebrafish by using CRISPR/*Cas9*.

**Fig. 6.**
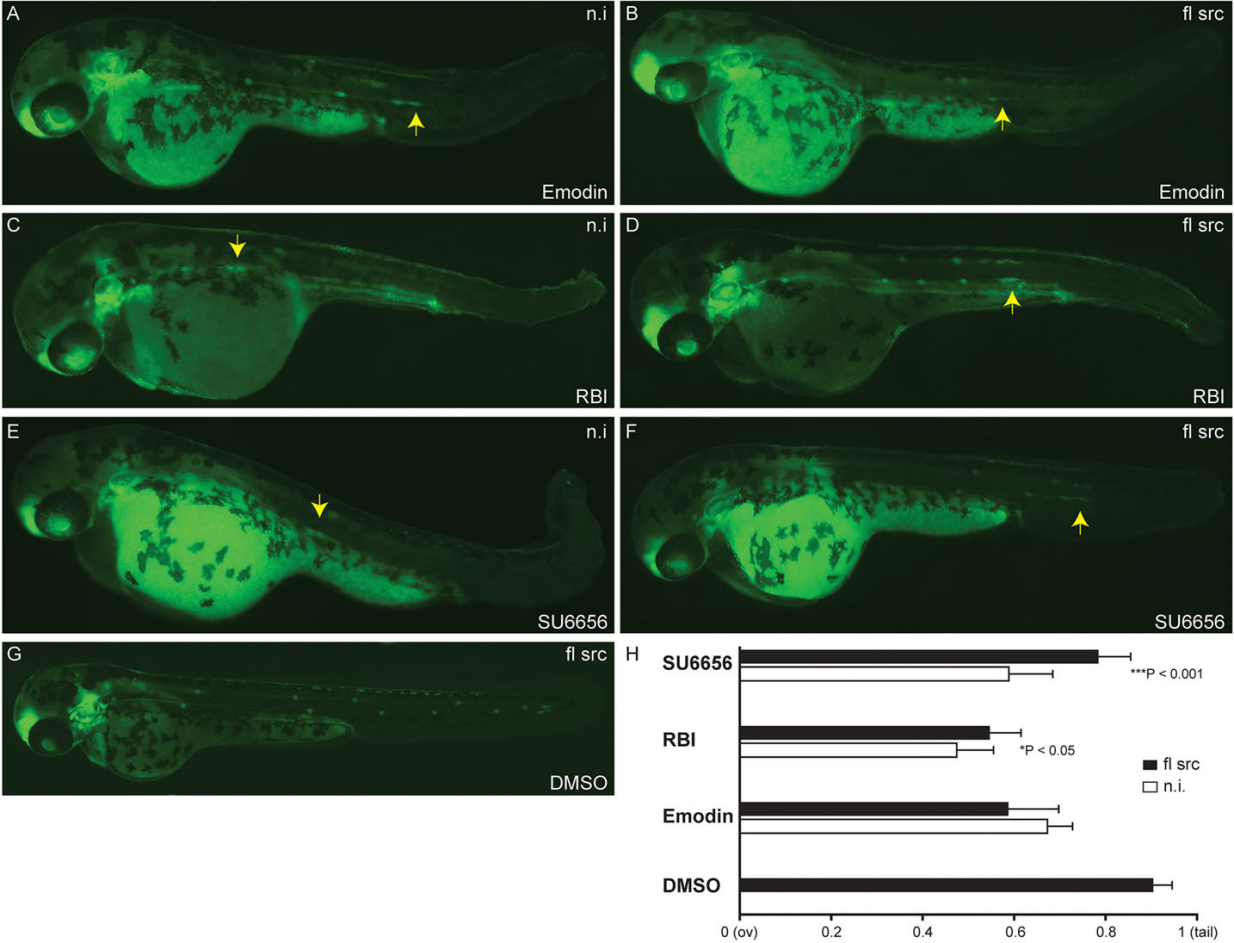
**Overexpression of *src* mRNA rescues PLLp migration disrupted by Src signaling pathway inhibitors.** Phenotypes of 48 hpf *cldnb:EGFP* embryos upon overexpression of *src* followed by treatment with Src inhibitors that had been identified in the screen. Overexpression of full-length (fl) *src* in embryos following DMSO treatment show a normal lateral line development (G). Overexpression of *src* partially rescued primordium migration defects in embryos treated with RBI (D) and SU6656 (F) compared with non-injected-embryos (C,E), respectively. This phenotype was not rescued in Emodin-treated embryos upon overexpression of *src* (A,B). (H) Quantification of the average primordium position in drug-treated embryos upon *src* overexpression relative to the distance from the otic vesicle (ov) to the tip of the tail. Experiments were carried out with ten larvae per condition. Error bars indicate +s.d. ***P<0.001; *P<0.05. mRNA (100 pg/2 nl) was injected into one-cell stage embryos. n.i., non-injected-embryo.

In addition, to avoid the off-target effects of Src inhibitors and validate the role of Src on PLLp migration, we also carried out rescue experiments (Fig. 6). Overexpression of *src* mRNA was able to partially rescue primordium migration defects induced by RBI (Fig. 6C,D) or SU6656 (Fig. 6E,F). Furthermore, overexpression of *src* reduces developmental abnormalities induced by these compounds. However, we did not see a significant rescue of phenotype in injected embryos treated with Emodin compared with WT (Fig. 6A,B). The possible explanation for this observation is that Emodin could be inhibiting other signaling pathways in addition to the Src family kinases involved in the PLL development preventing rescue (Shrimali et al., 2013; Wei et al., 2013). As expected, overexpression of *src* following DMSO treatment showed a normal lateral line phenotype (Fig. 6G) and was not able to rescue the LL phenotype induced by other signaling pathway inhibitors (data not shown).

### Inhibition of Src activity through SU6656 decreases tumor metastasis *in vivo*

**Fig. 7.**
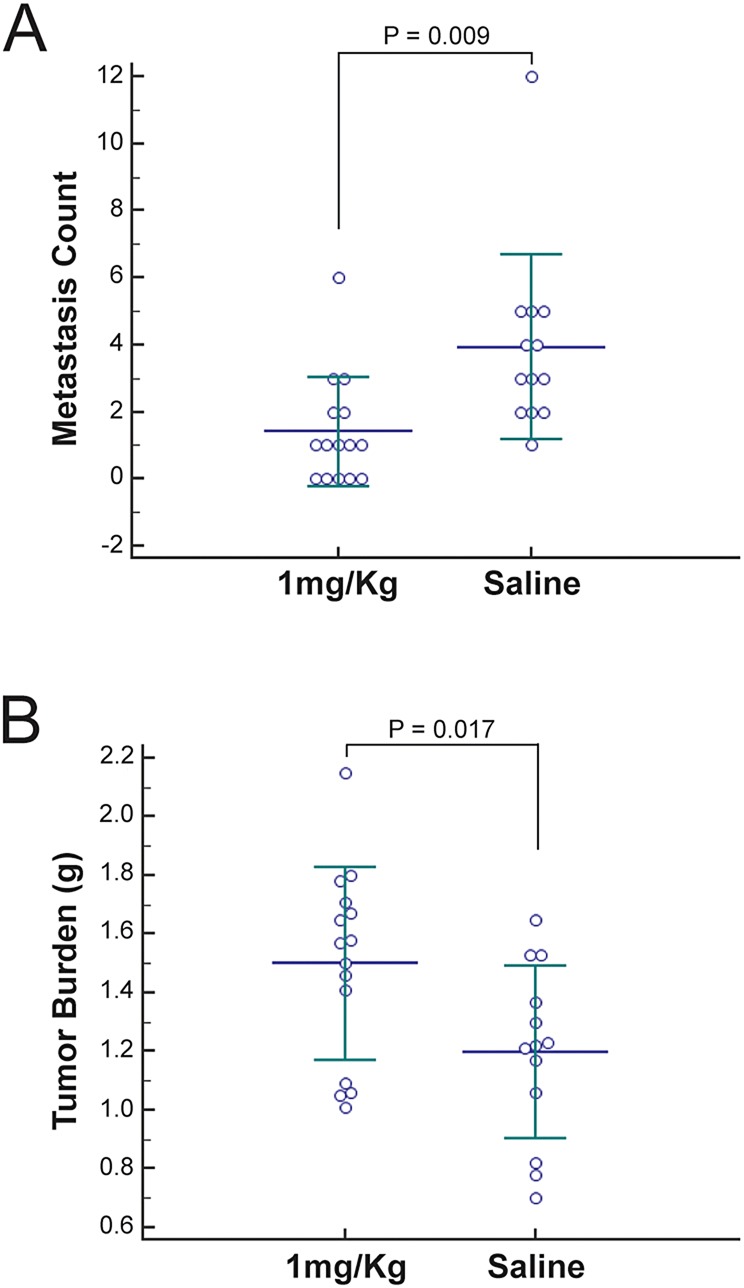
**Inhibition of Src activity by SU6656 decreases tumor metastasis *in vivo*.** After orthotopic implantation of metastatic cells into the mammary fat pad of BALB/cJ mice and 5 days of daily intraperitoneal injections of saline solution or 1 mg/kg SU6656, tumor burden at the site of injection and pulmonary metastases were assessed (saline, *n*=13; 1 mg/kg, *n*=15). Quantification as individual points for pulmonary surface metastases (A) and primary tumor burden (B) are shown. Both measurable differences are statistically significant, as indicated by *P*-values.

Key to the value of performing an *in vivo* screen in zebrafish for compounds that inhibit cell migration is to prove that identified compounds can inhibit cancer invasion *in vivo*. To demonstrate that inhibiting migration of the zebrafish PLLp can be used to identify anti-metastatic compounds we selected the Src inhibitor SU6656, which has a published effective pharmacologic dose in mice (Rehni and Singh, 2011; Rehni et al., 2012), for follow-up studies in a mouse tumor model. First, we tested the effect of SU6656 on the migration and proliferation of human 501-mel melanoma and mouse mammary tumor 4T1 cells in transwell assays. SU6656 impaired the migration of both cell lines in a dose-dependent manner, with EC_50_ values of 8.2 μM and 7.2 μM, respectively (supplementary material Fig. S3A). Likewise, SU6656 treatment reduced cell proliferation in a dose-dependent manner (supplementary material Fig. S3B,C). To evaluate the efficacy of this compound with respect to *in vivo* metastasis, orthotopic implantation of the highly metastatic 4T1 cell line into the mammary fat pad of female BALB/cJ mice was performed as previously described (Lee et al., 2014). Following orthotopic implantation of the cells, the mice were treated with daily intraperitoneal injections of saline or 1 mg/kg doses of SU6656 for 5 days. At 4 weeks post-implantation, tumor burdens were measured and pulmonary metastases were quantified. Mice treated with SU6656 displayed significant decreases in surface metastases compared with the saline-injected mice (Fig. 7A). This result not only confirmed the role of Src activity in tumor metastasis, but also the efficacy of Src inhibitor SU6656 to prevent metastases *in vivo*. This decrease in tumor metastasis was associated with a modest increase in primary tumor burden based on the average weight of the tumors (Fig. 7B). There are many possible explanations for this observation, including that SU6656 inhibits cells migrating to form metastases but does not have a major impact on overall cell viability. This experiment demonstrates that compounds or gene targets identified by inhibiting migration of the zebrafish PLLp can also have strong anti-metastatic activity in mammals *in vivo*.

## DISCUSSION

In the last 10 years, zebrafish has emerged as a leading model for drug discovery based on phenotype. New transgenes and powerful imaging technologies have increased the sensitivity and throughput to a degree that now makes it possible to screen thousands or even tens of thousands of compounds. Recent phenotype-driven screens have proven the efficacy of chemical screening using zebrafish, identifying small molecules that regulate signaling pathways, development and disease states (Jung et al., 2012; Sukardi et al., 2011; Taylor et al., 2010; Terriente and Pujades, 2013; Walker et al., 2012), whereas higher throughput, target-based screening on the whole has proven to be a disappointment (Swinney and Anthony, 2011).

To identify inhibitors of cell migration and, ultimately, anti-metastatic compounds, we developed a high-throughput-screening strategy combined with automated fluorescence microscopy to visualize the migration of the posterior lateral primordium. We took advantage of the *cldnb:EGFP* transgenic line, and tested the LOPAC^1280^, PKIS and NatProd collections to identify compounds that inhibited collective cell migration.

A total of 5.57% (*n*=165) of the compounds that we tested inhibited the formation of the posterior lateral line at concentrations from 0.5-10 μM, without being overtly toxic to the fish. However, it is possible that additional compounds would have inhibited migration if tested at concentrations higher than 10 μM, or might have inhibited motility at lower (sub-lethal) concentrations. Despite these caveats, the fact that almost 80% of the compounds tested were not toxic, and that a manageable number of compounds inhibited PLL formation suggests that 0.5, 1, 5 and 10 μM was an appropriate range of concentrations used in the screen.

As expected, a number of compounds previously described to contribute to tissue invasion and matrix remodeling were detected in our experiment (Chua et al., 2012; Quintavalle et al., 2011). *In vitro* high-throughput screening showed that inhibitors of Src (SU6656, RBI), MEK (UO126) and CDK (purvalanol A, GCP74514A, kenpaullone) all inhibited PLLp migration and, possibly, inhibited the formation of podosomes and/or invadopodia (Quintavalle et al., 2011).

To provide an independent verification of our results, we performed an *in vivo* inflammation assay based on neutrophil migration to sites of injury induced by noninvasive damage to lateral line neuromasts (d'Alencon et al., 2010). All the candidate drugs tested in this independent cell migration assay were confirmed to possess migration-inhibitory properties. Even though inflammation is based on the recruitment of individual neutrophils to the site of wounding or infection – and not coordinated and cohesive collective cell migration – in both individual and collective cell migration, cells need adhesion to, and traction on, the substratum, and progress through the extracellular matrix driven by distant guidance signals. These biophysical characteristics are regulated by similar molecular mechanisms and are induced through multiple signaling pathways.

Furthermore, to provide additional evidence for the success of our model, we performed *in vitro* migration/invasion assays using the human melanoma cell line 501-mel. Many of the tested compounds were able to inhibit the transwell migration of these cells, as well as Matrigel invasion but, notably, some compounds only appeared to inhibit migration *in vivo* and would have been missed had they been screened for using in an *in vitro* migration assay (supplementary material Table S3).

Src plays a critical role in a variety of cellular signal transduction pathways, regulating diverse processes such as cell proliferation, motility, adhesion, angiogenesis and survival (Kim et al., 2009). The proto-oncogene c-Src (Src) is a non-receptor tyrosine kinase whose expression and activity correlates with tumor progression, advanced malignancy and poor prognosis in a variety of human cancers (Chua et al., 2012; Homsi et al., 2007). Although the Src pathway is certainly already a heavily studied one in cancer research, it provided an excellent opportunity to demonstrate that zebrafish PLLp migration can be used to effectively identify anti-metastatic compounds that work *in vivo*. Three of the compounds detected in our screen are inhibitors of the Src pathway: Emodin, SU6656 and 7-cyclopentyl-5-(4-phenoxy)phenyl-7H-pyrrolo[2,3-d]pyrimidin-4-ylamine (RBI). In all cases, we observed a disruption in the migration of the PLLp. None of these compounds had been previously reported as an inhibitor of PLLp migration, nor had src activity been implicated in PLLp migration. However, recent studies have shown that the Src substrate and adaptor protein Tks5 plays a role in the migration of the zebrafish neural crest cells by generating actin-rich pro-migratory structures (Murphy et al., 2011) and is implicated in regulating invasive behavior of macrophages (Burger et al., 2011), suggesting that blocking Src has a general impact on cell migration. Moreover, the Src-Tks5 pathway regulates the formation of podosomes and invadopodia, degradation of the ECM, and the invasion of cancer cells *in vivo* and *in vitro* (Blouw et al., 2008; Courtneidge, 2012). Our data demonstrate that the Src-Tks5 pathway is directly involved in PLLp migration. Knockout of *src* and *tks5* through CRISPR/*Cas9* targeting resulted in a reduction of primordium size and loss of migration. Moreover, overexpression of *src* rescued the lateral line phenotypes induced by treatment with RBI or SU6656. Thus, taken together these results demonstrate that the Src pathway is the true target for these inhibitors and Src plays a role in PLLp migration.

We showed that the Src inhibitor SU6656 significantly reduced tumor metastasis in mice, demonstrating that similar mechanisms are used to control both cell migration during PLLp migration in zebrafish and cancer metastasis. These findings, collectively, support other investigations of Src and its binding protein Tks5, both as markers of invasive disease and as potential therapeutic targets (Arai et al., 2012; Blouw et al., 2008).

Although the demonstration that Src signaling is necessary for cell migration and metastasis may not be surprising, it demonstrates that the screen can effectively identify compounds with the desired anti-metastatic properties that will work *in vivo*. Therefore, our study provides a novel set of candidate molecules that block cell migration, and identifies (often surprising) target genes with potentially important roles in collective cell migration in both development and disease. These genes range from those involved in NO synthesis or neurotransmitter signaling, to unknown and potentially novel targets of natural compounds. Using this *in vivo* screening assay to screen libraries of pharmacologically active molecules of known bioactivity, or libraries designed to target specific classes of enzyme, such as kinases, allows rapid identification and direct testing of chemical targets *in vivo*. The new targeted inactivation of genes in zebrafish by using CRISPR/*Cas9* provides rapid and powerful confirmation of the bona fide target of the inhibitor. Using an *in vivo* model such as zebrafish instead of cell-culture systems will also allow for a more rapid and successful transition to pre-clinical mammalian models and, ultimately, to new chemotherapeutic treatments in humans.

In conclusion, we have shown that PLLp migration can be used as an effective model to rapidly identify potent compounds that comprise anti-metastatic activity, and the effectiveness of these compounds can be demonstrated in mammalian tumor models.

## MATERIALS AND METHODS

### Zebrafish husbandry

All animal experiments adhered to the NIH Guide for the Care and Use of Laboratory Animals. Zebrafish embryos and larvae of the *cldnb:EGFP* (Haas and Gilmour, 2006) and *Tg(mpx:GFP)* (Renshaw et al., 2006) strains were maintained in our facility according to standard procedures (Westerfield, 2000). All embryos were collected by natural spawning, staged according to Kimmel et al. (1995) and raised at 28.5°C in E3 medium (5 mM NaCl, 0.17 mM KCl, 0.33 mM CaCl_2_, 0.33 mM MgSO_4_ and 0.1% Methylene Blue) in Petri dishes, as described previously (Haffter and Nusslein-Volhard, 1996). Embryonic age is expressed in hours post-fertilization (hpf).

### Chemical library screening

The LOPAC1280 library (Sigma-Aldrich), composed of 1280 bioactive compounds, the NatProd library (MicroSource Discovery Systems Inc.), containing 800 pure natural products and their derivatives, and the GSK Published Kinase Inhibitor Set (PKIS), containing 880 compounds, were selected for the *cldnb:EGFP* embryo-based screenings. A total of 2960 compounds were screened. 200-nl aliquots of the chemicals (10 mM in DMSO) were transferred by ultrasonic deposition using an ATS-110 (EDC Biosystems) from the mother plates into 96-well culture plates (Costar #3720) to generate diluted stock plates (200 µl final volume). Next, after manual removal of the chorions, *cldnb:EGFP* embryos were arrayed manually into the 96-well plates (two embryos per well) in 200 μl of system water at 20 hpf, resulting in a final screening concentration of 10 µM. The positive control for this assay was the c-Met inhibitor K252a (Calbiochem #420298; 0.01 to 10 µM), and the negative control was 1% DMSO. Embryos were treated with the compounds at room temperature over 2 days. At 36 and 48 hpf, embryos were anesthetized with 0.016% Tricaine, and a fluorescence image of individual wells was taken automatically (magnification 4×) using an iCys research imaging cytometer (Molecular Devices). Images were captured and compiled for visual inspection. Drugs were considered to be active when the two embryos displayed the same phenotype (edema, lethality or other phenotypes); all putative hits were retested and comparable results were obtained in each case.

### Secondary chemical screening

The compounds that induced developmental toxicity effects and caused embryo death in the primary screening were re-tested in *cldnb:EGFP* embryos by using final screening concentrations of 0.5, 1 and 5 μM. Screening procedures were carried out as described above.

### Confirmatory tests

In follow-up studies, 20 compounds from the LOPAC1280 and the NatProd libraries that induced lateral line phenotypes were tested in *cldnb:EGFP* embryos. Chemical treatment of embryos (*n*=15 per well) was initiated at 20 or 36 hpf and finished at 48 hpf. Embryos were then anesthetized with 0.016% Tricaine and fluorescence images of embryos were taken using an inverted Zeiss AXIOVERT200M microscope equipped with an Apotome grid confocal system.

### Chemically induced inflammation assays

Chemically induced inflammation (ChIn) assays were performed as described in d'Alencon et al. (2010). Briefly, 15 transgenic *Tg(mpx:GFP)* larvae at 72 hpf were transferred to 12-well plates in a volume of 1 ml of E3 solution. For inhibition, test reagents were added to reach the required concentration to the well. Positive and negative control larvae were incubated with 1% of DMSO. Incubation with DMSO control and tested drugs was carried out for 30 min prior to addition of 10 μM CuSO_4_; the latter was added directly to the wells that contained the experimental and control larvae for 40 min at 28°C. Larvae were then fixed with 4% paraformaldehyde prepared in phosphate-buffered saline (PBS) and incubated for 1 h at room temperature. After fixation, larvae were washed three times for 5 min each in PBS-Tween20 with gentle agitation. Counting of leukocytes was done using an inverted Zeiss fluorescence microscope.

### Cell culture

501-mel human melanoma cells were cultured in DMEM (Invitrogen) supplemented with 10% fetal bovine serum (Invitrogen), antibiotic-antimycotics (100 U/ml penicillin, 100 µg/ml streptomycin, 250 ng/ml amphotericin; Invitrogen) and 2 mM L-glutamine (Invitrogen).

### Transwell migration assay

Cell motility was determined *in vitro* using a Transwell chamber (BD Biosciences) according to the manufacturer's instructions. Briefly, 8-mm pore cell-culture inserts were rehydrated for 2 h in 37°C serum-free DMEM prior to use. Then 2.5×10^5^ 501-mel human melanoma cells were placed on the upper chamber in 500 μl serum-free DMEM plus 0.1% DMSO control or a test drug. In the lower chamber, 750 μl DMEM plus 10% FBS was added. After 16 h of incubation at 37°C in 5% CO_2_, cells from the upper surface of the membranes were removed by gentle swabbing, and the cells that had migrated through the pores were fixed, stained and counted. Relative migration was based on the average number of cells on the underside of the membrane in five random images generated at 200× magnification per chamber of two independent experiments, and was normalized to the results from DMSO control cells.

### Cell invasion assay

Invasion of cells into Matrigel was determined *in vitro* using 24-well BioCoat Matrigel inserts (Becton Dickinson) as described in McGill et al. (2006). Briefly, the invasion chambers were prehydrated with serum-free DMEM (500 μl/well) for 2 h of incubation at 37°C in 5% CO_2_. After trypsinization, 501-mel melanoma cells (5×10^5^) were suspended in 500 μl serum-free medium and incubated with 0.1% DMSO control or different inhibitors for 20 min, and then placed in the upper compartment of the plates. Subsequently, the lower compartment was filled with 10% FBS medium (750 μl) including recombinant human HGF (50 ng/ml). After 24 h of incubation, cells were fixed, stained and counted. Non-migratory cells on the upper filter surface were removed using a cotton swab, and the total number of invasive cells was counted at 200× magnification using a phase-contrast microscope. Relative invasion was based on the average number of cells on the underside of the membrane in five random images of two independent experiments, and was normalized to the results from DMSO-control cells.

### Targeting of *src* and *tks5a* genes with the CRISPR/Cas9 system

We generated *src* and *tks5a* knockouts using the CRISPR/Cas9 targeting system, by targeting two different exons in each gene. The targeting sequences were as follows: *src* target 1: 5′-GGATTTCCTGAAAGGTGACA-3′, *src* target 2: 5′-GGCACCGTCTGACTCCATCC-3′, *tks5a* target 1: 5′-GGTGCTGGAGCAGTACGTGG-3′, *tks5a* target 2: 5′-GGAGATGTGGCGCTCAGGGG-3′.

Single-guide RNAs (sgRNAs) were synthesized by annealing and extending two oligonucleotides, and mRNA was transcribed from assembled oligonucleotides using the T7 RNA synthesis kit (Varshney et al., 2015). The *cas9* mRNA was prepared by *in vitro* transcription from the pT3TS-nls-zCas9-nls plasmid (Jao et al., 2013). We co-injected two sgRNAs (25 ng/µl) and 300 ng/µl Cas9 in one-cell stage *cldnb:EGFP* embryos. Injected embryos were raised to sexual maturity. The founder fish were out-crossed with wild-type fish, and the germline mutations were identified using fluorescence PCR (Sood et al., 2013) and sequencing. Phenotypes were also scored in some embryos 48 h after injection of CRISPR/Cas9-targeting RNAs.

### Microinjection of synthetic *src* mRNA, rescue experiments

100 pg of mRNA in water was injected into one-cell stage *cldnb:EGFP* embryos. We used full-length *src* cDNA (Clone ID: 8104558, Dharmacon) and capped synthetic mRNAs were prepared using the T7 mMessage mMachine kit (Ambion). For rescue experiments, chemical treatment of *src*-mRNA-injected embryos (*n*=10 per well) with the Src inhibitors Emodin, RBI or SU6656, or with a DMSO control, was initiated at 20 hpf and finished at 2 dpf. Embryos were then anesthetized with 0.016% Tricaine, and fluorescence images of embryos were captured using an inverted Zeiss AXIOVERT200M microscope and quantified for PLLp migration.

### Orthotopic mammary fat pad injections and SU6656 treatment

Female BALB/cJ mice were purchased from Jackson Laboratory (Bar Harbor, ME). Injections of the mouse mammary tumor 4T1 cells were performed as previously described (Lee et al., 2014). Briefly, 10^5^ cells in 100 μl saline solution were orthotopically implanted into the mammary fat pads of 10- to 12-week-old female BALB/cJ mice. Beginning on the same day as the implantations, the mice received daily intraperitoneal injections of either saline solution (*n*=13) or 1 mg/kg SU6656 (*n*=15) for 5 days. SU6656 dosage was determined based on previous publications (Rehni and Singh, 2011; Rehni et al., 2012). Three weeks after the implantations, mice were euthanized. Tumors were dissected and weighed. Lungs were isolated and surface metastases enumerated by eye using a dissecting microscope, as previously described (Crawford et al., 2007). All animal experiments were performed in compliance with the National Human Genome Research Institute Animal Care and Use Committee's guidelines.

### Statistical analyses

Results from PLLp migration assays, *in vivo* ChIns, *in vitro* migration and invasion cell assays, and *in vivo* mouse cancer invasion assays are represented as mean±s.d. of independently performed assays. Statistical analysis was performed by comparing means of biological replicates using the unpaired two-tailed Student's *t*-test (Excel, Microsoft). A value of *P*<0.05 was considered as significant.

## Supplementary Material

Supplementary Material
