# Supplementary material for: Phenotype-driven chemical screening in zebrafish for compounds that inhibit collective cell migration identifies multiple pathways potentially involved in metastatic invasion
Source: Dis Model Mech. 2015 Jun 1;8(6):565–76. doi: 10.1242/dmm.018689 (PMC4457032; doi:10.1242/dmm.018689)
Supplement: Supplementary Material [file supp_8_6_565__index.html]

Phenotype-driven chemical screening in zebrafish for compounds that inhibit collective cell migration identifies multiple pathways potentially involved in metastatic invasion — Supplementary Material 

# Phenotype-driven chemical screening in zebrafish for compounds that inhibit collective cell migration identifies multiple pathways potentially involved in metastatic invasion

## DMM018689 Supplementary Material

**Files in this Data Supplement:**

- **Supplementary Material**
