# Supplementary material for: Phenotype-driven chemical screening in zebrafish for compounds that inhibit collective cell migration identifies multiple pathways potentially involved in metastatic invasion
Source: Dis Model Mech. 2015 Jun 1;8(6):565–76. doi: 10.1242/dmm.018689 (PMC4457032; doi:10.1242/dmm.018689)
Supplement: Supplementary Material [file supp_8.6.565_DMM018689.pdf]

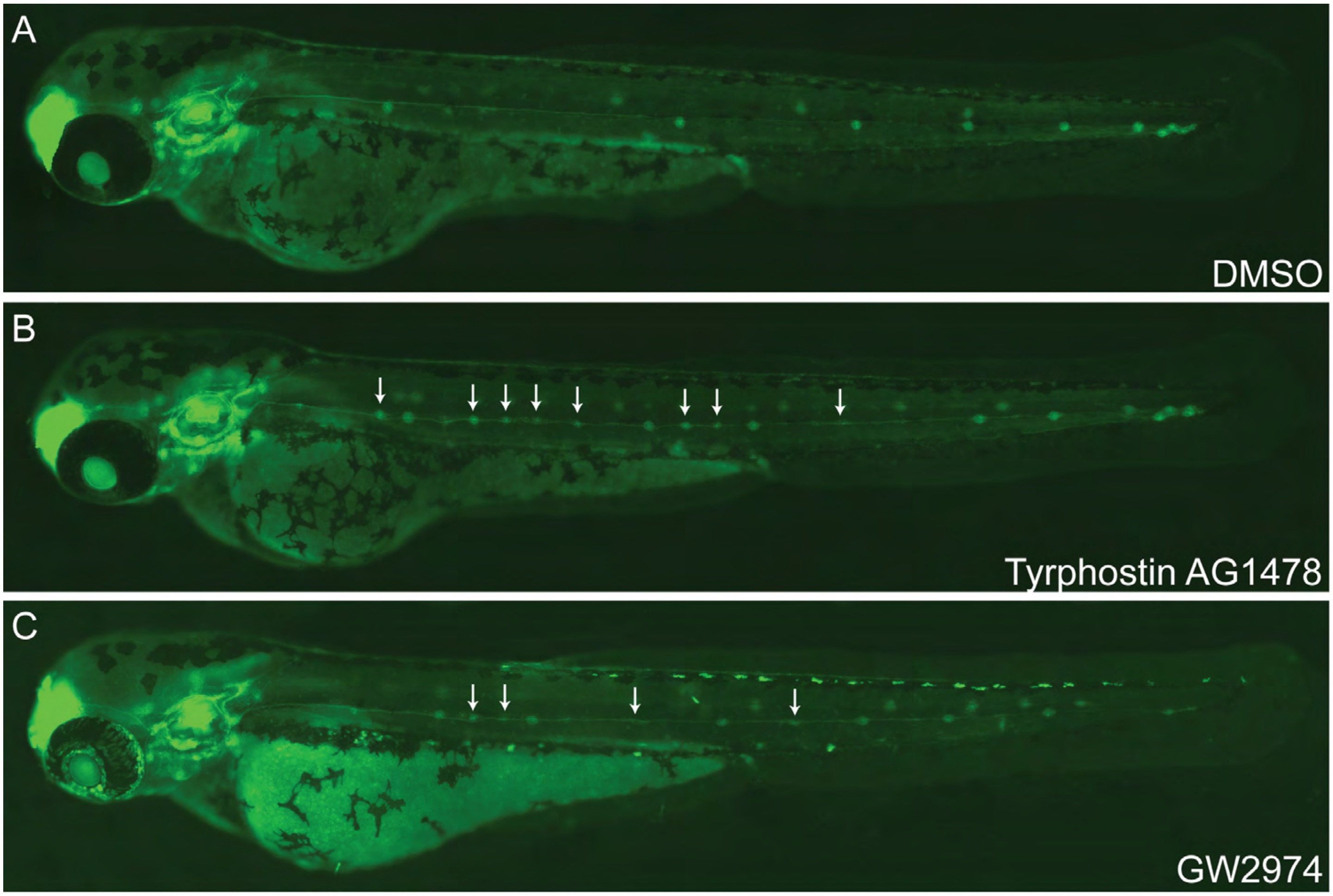

Supplemental figure 1. Regulation of neuromast number by ErbB signaling.

Phenotypes of 48 hpf zebrafish embryos treated with ErbB signaling pathway inhibitors showed an unusual increase in neuromast numbers. Treatments of 20 hpf *cldnb:EGFP* embryos with 10μM of Tyrphostin AG1478 (B) and GW2974 (C) increased the number of deposited neuromasts compared to DMSO control (A). Arrow indicates the aberrant organization of interneuromast cells in treated embryos as compared to control.

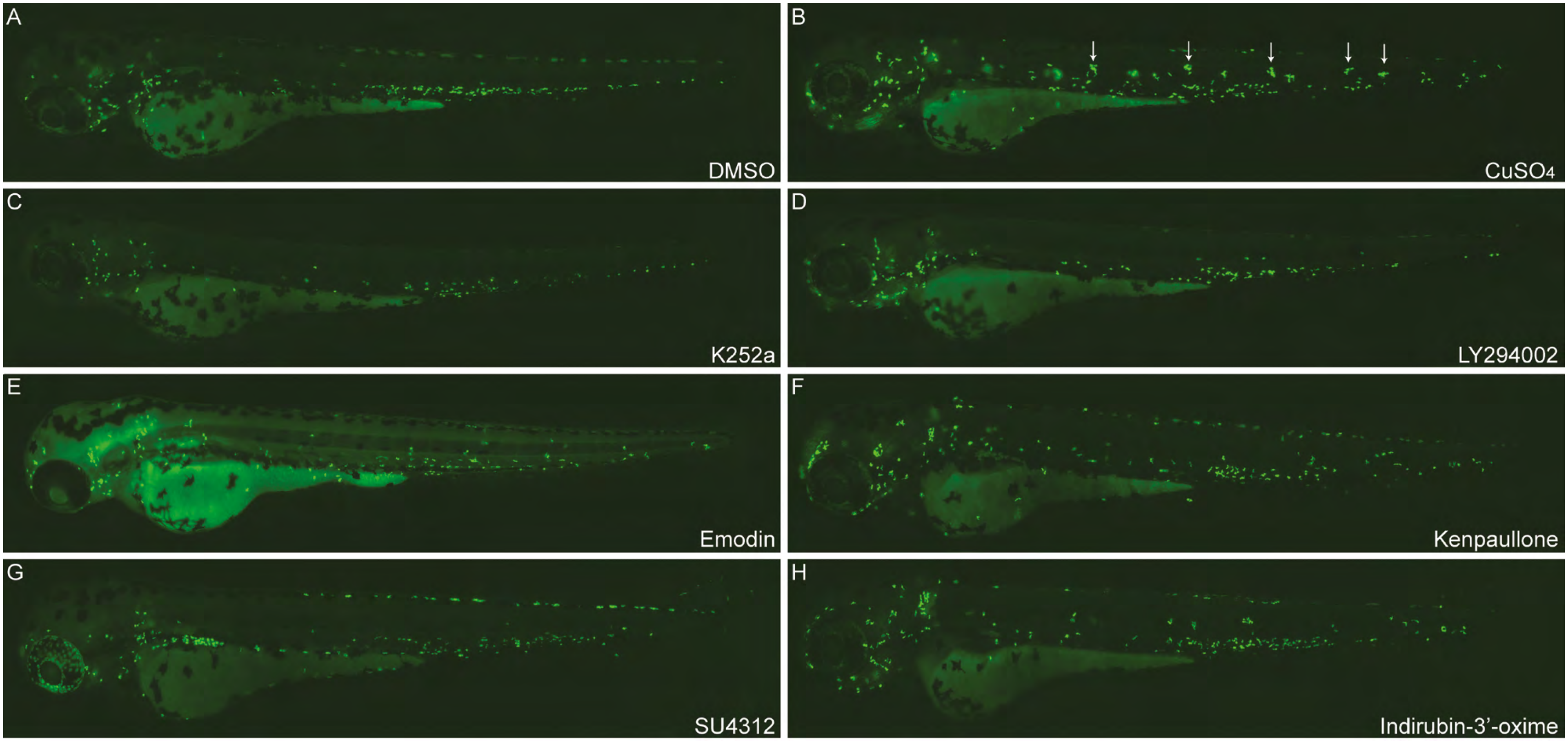

Supplemental figure 2. Chemically induced inflammation (ChIn) assay.

DMSO-treated *Tg(mpx:EGFP)* larvae show the normal distribution of labeled cells, mostly localized in the ventral trunk and tail (A). In copper-treated siblings (B), leukocytes become localized preferentially to a few clusters along the horizontal midline of the trunk and tail (white arrows). Larvae exposed to indicated compounds (C-H) exhibit a decreasing number of leukocytes along the PLL. Inhibitors (C-H) were added to the incubation medium 30 min prior to addition of copper and were tested for inhibition of leukocyte migration using ChIn assays.

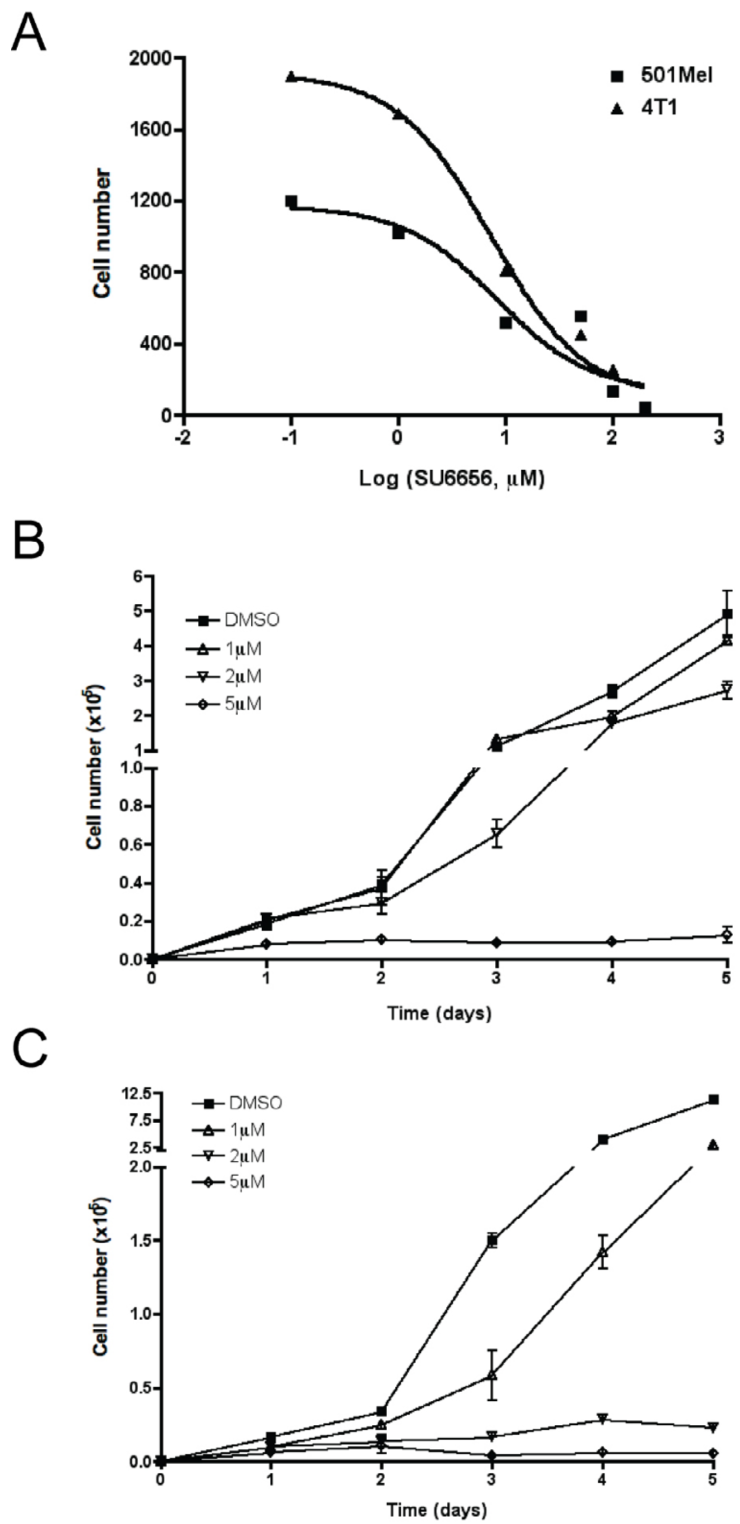

Supplemental figure 3. SU6656 inhibits cell motility and proliferation of cancer cells *in vitro*.

(A) EC50 measurements for cell migration inhibition. Human 501Mel melanoma and mouse mammary tumor 4T1 cells were cultured in the presence of SU6656 at indicated concentrations, or DMSO as a negative control for 16 hours; and then assayed for motility. EC50 values were calculated from the dose-response curves using GraphPad Prism software. (B-C) Growth curves of human 501Mel melanoma (B) and mouse mammary tumor 4T1 (C). Cells were cultured in the presence of SU6656 (1, 2 or 5 μM) or DMSO control and counted at the indicated times.

Sup Table 1: Summary of targeted sites with their mutagenesis activities

Download Table S1

Sup Table 2: Total number of unique alleles per gene

Download Table S2

Sup Table 3: Summary of germline mutagenesis data from multiplex gene targeting

Download Table S3
